# Acute effects of Square Stepping Exercise on cognitive and social functions in sedentary young adults: a home-based online trial

**DOI:** 10.1186/s13102-021-00309-w

**Published:** 2021-08-02

**Authors:** Masato Kawabata, Su Ren Gan, Gint Goh, Siti Aisha Binte Omar, Ivan T. F. Oh, Wan Qi Wee, Tomohiro Okura

**Affiliations:** 1 Nanyang Technological University, National Institute of Education, 1 Nanyang Walk, 637616 Singapore, Singapore; 2 School of Human Movement and Nutrition Sciences, The University of Queensland, St Lucia, QLD 4072 Australia; 3 Faculty of Health and Sport Sciences, University of Tsukuba, 1-1-1 Tennodai , Tsukuba, Ibaraki 305-8574 Japan

**Keywords:** COVID-19, Executive function, Group cohesion, Integrated exercise, Online intervention

## Abstract

**Background:**

The Square Stepping Exercise (SSE) is an exercise training program incorporating cognitive and physical exercise components, which was originally developed for older adults to reduce falling risks. SSE’s potential in delaying cognitive decline in older adults seems to be promising. However, there is scarce research on the SSE program with young adults. Furthermore, the outbreak of coronavirus disease has imposed people to change their lifestyle and behaviors, including exercise behaviors. Hence, the purpose of this study was to examine the acute effects of a home-based online SSE trial on cognitive and social functions in sedentary young adults.

**Methods:**

A total of 18 young adults (6 males, 12 females) participated in the present study. They completed two exercise conditions (SSE and active control exercise), consisting of 3 sessions per week, over 2 weeks. A 2 times (pre vs. post) × 2 conditions (SSE vs. active control) repeated-measures ANCOVA was conducted on the score of the Modified Card Sorting Task with age and education year as covariates. A one-way repeated-measures MANOVA was performed on the subscale scores of the Physical Activity Group Environment Questionnaire to examine the effects of the exercise conditions (SSE vs. active control) on group cohesion.

**Results:**

SSE was found effective to improve executive function such as abstract reasoning, mental flexibility, and problem-solving skills. Furthermore, participants’ perceptions of social interaction with their group, and closeness and bonding existing in their group were significantly higher in the SSE condition than the active control condition.

**Conclusions:**

In the present study, SSE was conducted online and found to be effective to enhance executive function and group cohesion in sedentary young adults. These novel approach and findings are the strengths of the present study. People aged 60 years and over are more vulnerable to the coronavirus and at higher risk of developing serious illness. Given the coronavirus pandemic circumstances, it is worthwhile to explore the possibility of the online SSE approach to older adults in future research.

## Background

Cognitive and physical exercise done independently can enhance cognition in both cognitively normal and cognitively impaired individuals. A review of such interventions [1] has suggested that combining both cognitive and exercise training in an intervention program may be advantageous to increase this enhancement. This type of activity has yet to be well researched and implemented.

One of the training programs that incorporate both cognitive and physical exercise components is the Square Stepping Exercise (SSE) [2]. SSE is a form of stepping exercise training that can be easily conducted in a group setting without high cost of materials and equipment. It was developed for older adults to do exercise indoors by overcoming challenges faced when walking outdoors. Furthermore, SSE has the potential to promote social interactions when it is conducted in a group setting [2].

Initial studies of 12-weeks SSE program conducted in Japan revealed that SSE was effective to reduce fall risks in healthy community dwelling older adults [2–4]. Ravichandran et al. [5] reported that a 4-week SSE program was useful to improve balancing and gait abilities of older adults with Parkinson’s disease. These findings are corroborated by recent meta-analytic reviews of the effects of SSE on fall prevention [6, 7] and it was shown that SSE is effective in preventing the risks of falling by improving balance. The SSE was also found to improve older adults’ depressive symptoms [8] and cognitive functions [9–11]. Teixeira et al. [9] reported that a 14-week SSE program was effective to improve global cognition, attention and mental flexibility in Brazilian older adults. In addition, cognitive gains in memory and executive function were found in Japanese older adults [10, 11].

Acute versus chronic exercise is one of the very important distinctions concerning the protocols to study the effect of exercise on cognition and physical functions and they need to be distinguished [12]. The Quadrato Motor Training (QMT), developed by Paoletti [13], is whole-body movement training with oral instructions (e.g., a specifically-structured walking). This is another exercise training program that incorporates both cognitive and physical exercise components. Ben-Soussan and colleagues conducted a series of studies to examine both acute and chronic effects of the QMT on cognitive functions in healthy young adults [14–16]. For the acute effects of the QMT, they found that a short (7 min) QMT session induced cognitive improvements (e.g., decreased reaction time, increased ideational flexibility), as opposed to the two other intervention conditions such as a simple motor training and verbal training [14, 15].

Both SSE and QMT include stepping movements. According to the results of the above studies on the chronic effects of the SSE program, SSE’s potential in reducing falling risks and delaying cognitive decline in older adults seems to be promising. However, there is scarce research on the SSE program with young adults as SSE was developed for older adults. Furthermore, no one has investigated the acute effects of SSE on cognitive functions. Hence, the primary purpose of the current study was to examine the acute effects of SSE on cognitive functions in sedentary young adults.

The outbreak of coronavirus (COVID-19) disease has imposed people to change their lifestyle and behaviors. When participating in a group exercise, people have been requested to keep a safe distance from others and reduce physical interactions to prevent infection. The SSE program was conducted online in this study to maximize the safety of participants. Therefore, the secondary purpose of the study was to investigate if group cohesion would be enhanced through a home-based SSE trial conducted online.

## Methods

### Participants and procedures

A sample of 18 young adults (6 males, 12 females; *M*_age_ = 22.80 years, *SD* = 1.17; *M*_body mass index_ = 22.04 kg/m^2^, *SD* = 3.80) participated in the present study. Participants’ physical characteristics are summarized in Table 1. Participants were recruited in Singapore with the following inclusion criteria: (a) aged between 21 and 29 years old; (b) exercise less than three times per week and not more than 30 min each time; and (c) no psychiatric or neurological disorders.

**Table 1:** Physical characteristics of participants (*N* = 18)

	Male (*n* = 6)	Female (*n* = 12)	Total (*N* = 18)
Age (years)	23.50 (± 1.52)	22.40 (± 0.79)	22.80 (± 1.17)
Height (m)	1.74 (± 0.07)	1.63 (± 0.06)	1.66 (± 0.08)
Weight (kg)	76.12 (± 11.19)	53.70 (± 5.28)	61.17 (± 13.15)
BMI (kg/m^2^)	25.29 (± 3.90)	20.41 (± 2.60)	22.04 (± 3.80)

*Note*. Data are *Mean* (*SD*)

Prior to the data collection, a statistical power analysis was conducted using G*Power version 3.1.9.4 [17] to determine appropriate sample size for a repeated-measures ANOVA. Based on the estimated effect sizes for the effect of SSE on cognitive functions (*f* = 0.29; Teixeira et al. [9]), it was estimated that 18 participants would be required to achieve a power of 0.80 at the alpha level of 0.05 [18] for the repeated within-subjects analysis of 1 group and 4 measurement time points (2 measurement time points in two conditions). Ethical approval was obtained from an Institutional Review Board before all testing. Participation was voluntary and informed consent was received from each participant. This study was conducted according to the guidelines and procedures involving human subjects, which were approved by the Institutional Review Board.

**Fig. 1:**
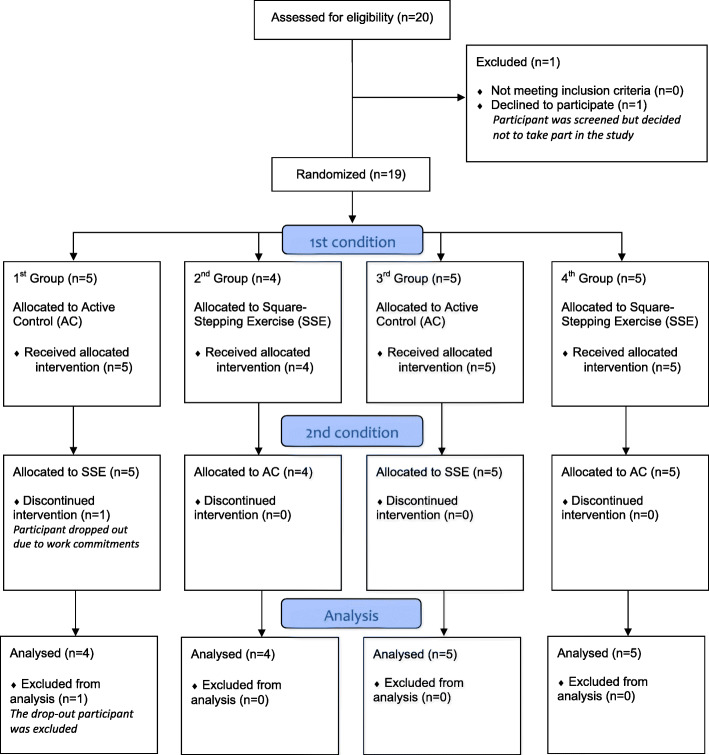
Flow of study design

A counterbalanced cross-over design with two different conditions (SSE and active control exercise) was employed. A pair of researchers conducted every exercise session. One led the session as the main instructor and the other supported the main instructor as a sub-instructor. They rotated their roles in every session. To minimize the waiting time in the SSE condition, participants were randomly divided into four groups of four to five individuals based on their availability for exercise sessions. The sequence of the groups undergoing either of the conditions was counterbalanced to minimize the effect of the previous exercise condition on the other (see Fig. 1). That is two groups underwent the SSE first and then the active control exercise, whereas the other two groups underwent the exercises in the opposite sequence. The interval between the two exercise conditions was 2–4 days across the four groups. The exercise sessions for both conditions were conducted online through Zoom, an online video conferencing software, and were scheduled in the evening. For each group, every session was conducted at the same time to minimize the effect of time of day on the outcome variables. All participants were required to attend three sessions per week over two weeks, and they participated in every session. Each exercise session comprised of 5 min of warm-up activities, 30 min of the main exercise (SSE or active control exercise), and 15 min of cool-down.

### Exercise protocol

#### Square Stepping Exercise

**Fig. 2:**
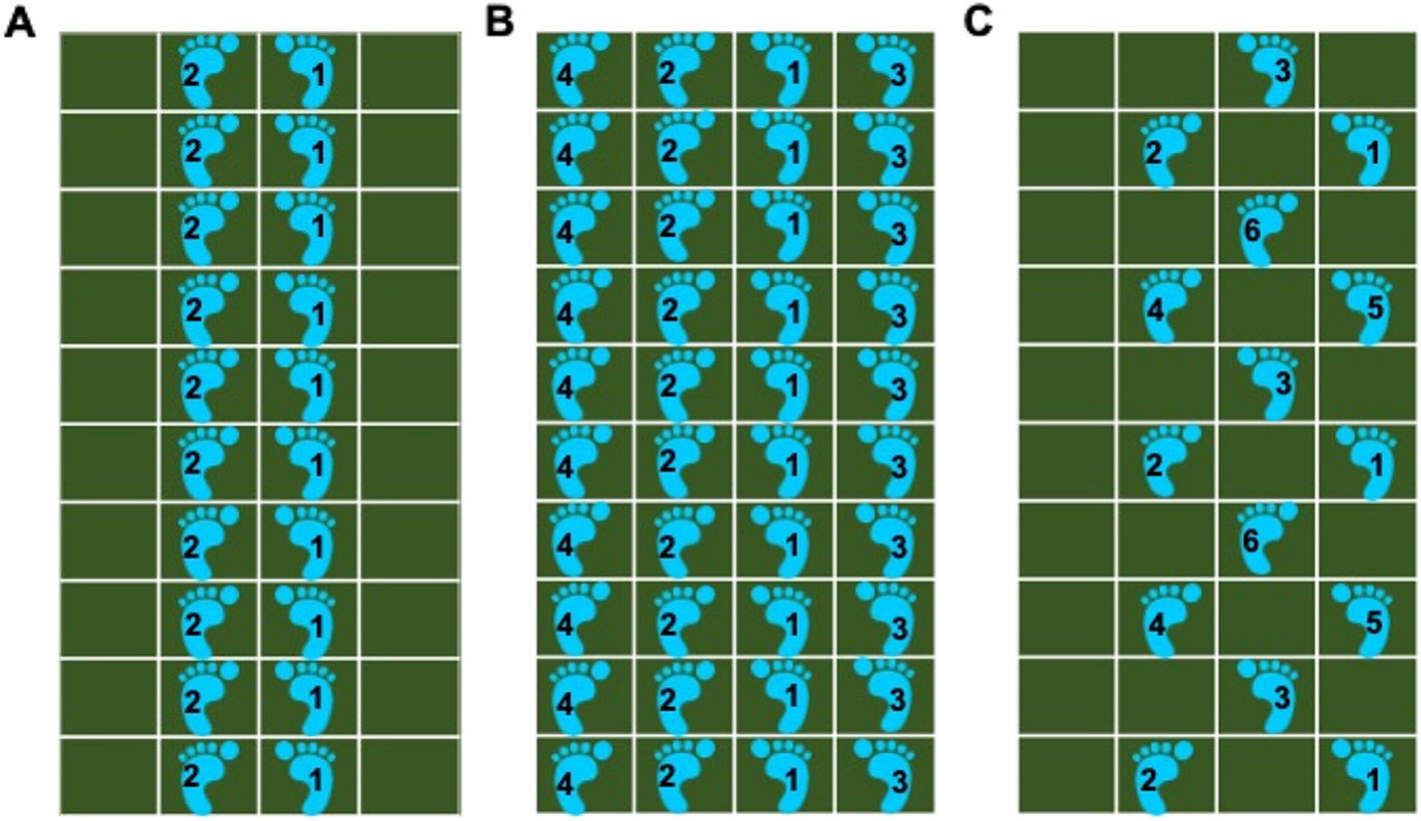
Sample of SSE patterns of varying difficulties, from left to right, **A** Beginner level, pattern 3, **B** Intermediate level, pattern 1, and **C** Intermediate level, pattern 7. (adapted from https://square-step.org/)

SSE was carried out on a thin mat (100 cm × 250 cm) that was partitioned into 40 squares (25 cm each). Participants received the SSE mats from the researcher prior to the SSE condition. They were advised to set the video camera device at a good location so that the instructors can see the entire mat and evaluate participant’s performance clearly. The SSE program involved multi-direction movements including forward, backward, lateral and oblique step patterns (see Shigematsu et al., [2, 3] for the details of SSE). The SSE patterns are categorized into 3 levels (elementary, intermediate, and advanced) based on the complexity of the stepping patterns. From the list of SSE patterns developed by the Institute for Square Stepping Exercise [19], 25 patterns were selected for this study: 8 elementary, 8 intermediate, and 7 advanced patterns. Figure 2 indicates a sample of three SSE patterns of different level of difficulty.

An instructor demonstrated a stepping pattern to participants at the beginning of each round. Participants were required to memorize the pattern demonstrated. Subsequently, they stepped from one end of the mat to the other by following the stepping pattern demonstrated by the instructor. They had to complete each pattern accurately two to three times before proceeding to a more complex step pattern. The other instructor evaluated the participant’s movements for accuracy. Participants took turns to perform the pattern introduced in each round. Once completed, they returned to the starting position by walking outside the mat. While waiting for their turn, participants were instructed to walk on the spot to ensure that they were constantly moving.

On average, three to five stepping patterns were taught in each SSE session. Every participant was required to pass the assessment round in order for the group to progress through the patterns. The criteria for passing each assessment were: (a) accuracy of the footsteps, which is accurately placing the entire ball and the toe of the foot within the demarcated boxes on the SSE mat, (b) a knee raise, which is perpendicular to the body were the main criteria for passing, and (c) correct placement of the feet, which is remembering and performing the correct pattern of steps.

 To simulate the social conditions of a face-to-face SSE session, interactions between participants were promoted in the online SSE session. Each participant was randomly assigned another participant as their partner in each session, and participants made a pair with a different partner on all three sessions. The partners were required to provide encouragements through the provision of virtual hi-fives and words of encouragements whenever their partners have completed a round of the SSE exercise.

#### Active control exercise

Walking on a spot at a similar intensity level was used as an active control exercise. The active control exercise was employed in this study to clarify that differences between the two conditions were due to the cognitive and social aspects of the SSE rather than physical aspects of the exercise. The intensities of the SSE and the active control exercise were preliminarily compared among four authors by measuring the heart rate during both exercises and it was found that exercise intensities were similar to each other (SSE: 58–75 bpm; Active control: 59–77 bpm).

In the active control condition, the main instructor demonstrated knee raise and lateral stepping to mimic the physical bodily movements of SSE. The instructor’s screen was spotlighted on the Zoom platform. Participants were required to simply follow the demonstrated movements on the same spot without any interaction between group members. The movements in the active control condition are listed in Table 2. Participants repeated three sets of the movements in every session.

**Table 2:** Movements in the active control condition

Flow of the Movements	Duration
1. Marching on the spot	90 s
2. Walking on the spot	30 s
3. Side-to-side stepping	90 s
4. Walking on the spot	30 s
5. Knee-to-chest stretching (2 counts hold)	90 s
6. Walking on the spot	30 s
7. Hip circumduction (outwards & inwards)	90 s
8. Walking on the spot	30 s
9. Reaching up and marching on the spot	90 s
10. Short break	30 s

### Measures

#### Executive function

The Modified Card Sorting Task (MCST) [20] was carried out by using Inquisit 5 [21] to measure executive function such as abstract reasoning, mental flexibility, and problem-solving skills. The MCST scoring is based on the number of categories completed and the total number of perseverative errors. The total number of perseverative errors was used as a primary MCST score, according to Caffarra et al.’s [22] recommendation. The test was administered to participants before and after the second and third sessions of both the exercise conditions. Participants could familiarize themselves with the test sufficiently through practice in the second session. This approach was employed to attenuate practice effects on the MCST data in the third session [23]. Therefore, only the MCST data collected in the third session of both conditions were used for subsequent data analysis.

#### Group cohesion

The Physical Activity Group Environment Questionnaire (PAGEQ) [24] was used to measure participant’s perceptions of cohesion in their exercise groups. The PAGEQ is a 21-item instrument, consisting of the four dimensions: Individual Attractions to the Group—Task (ATG-T: personal involvement with the group task), Individuals Attractions to the Group—Social (ATG-S: personal acceptance and social interaction with the group), Group Integration—Task (GI-T: the closeness and bonding that exists within the group as a totality around its collective task), and Group Integration—Social (GI-S: the closeness and bonding that exist within the group as a totality around social concerns). Participants were asked to indicate the degree to which they agreed with the statement of each item on a 9-point Likert-type scale, ranging from 1 (*very strongly disagree*) to 9 (*very strongly agree*). The PAGEQ was completed by participants immediately after the last session of each exercise condition.

### Data analysis

A 2 times (pre vs. post) × 2 conditions (SSE vs. active control) repeated-measures ANCOVA was conducted on the MCST score with age and education year as covariates [22]. A one-way repeated-measures MANOVA was performed on the PAGEQ subscale scores to examine the effects of the exercise conditions (SSE vs. active control) on group cohesion.

## Results

### Executive function

**Fig. 3:**
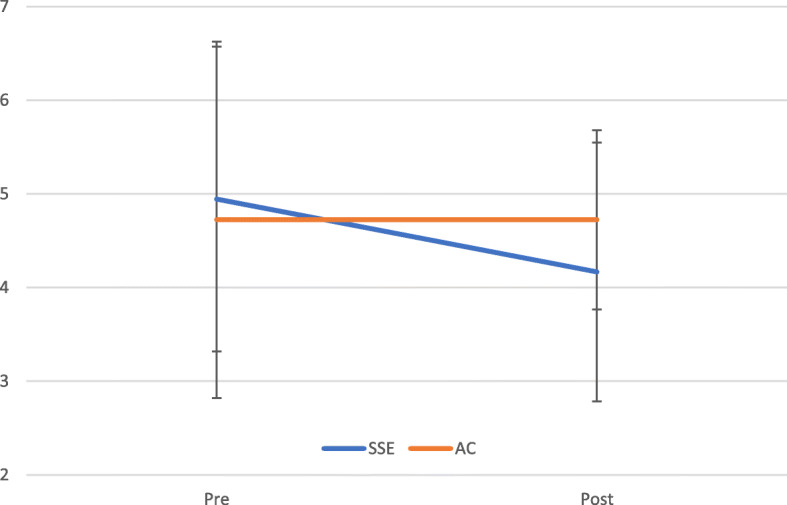
The total number of the Modified Card Sorting Task (MCST) in Square Stepping Exercise (SSE) and Active Control (AC) conditions. Error bar: *SD*

A 2 times (pre vs. post) × 2 conditions (SSE vs. active control) repeated-measures ANCOVA on the total number of the MCST perseverative errors revealed that none of the main and interaction effects were significant (see Fig. 3 for descriptive statistics of the value). Considering both conditions were exercise conditions with similar physical intensity and bodily movements, these non-significant effects were understandable. To examine the score in more detail, a paired *t*-test was separately conducted for each condition. It was found that the total number of the perseverative errors (pre: *M* = 4.94, *SD* = 1.62; post: *M* = 4.17, *SD* = 1.38) was significantly reduced in the SSE condition, *t*(17) = 2.72, *p* = .015, *d* = 0.1.32, whereas the score was unchanged before and after the exercise session in the active control condition (pre: *M* = 4.72, *SD* = 1.90; post: *M* = 4.72, *SD* = 0.96).

### Group cohesion

**Fig. 4:**
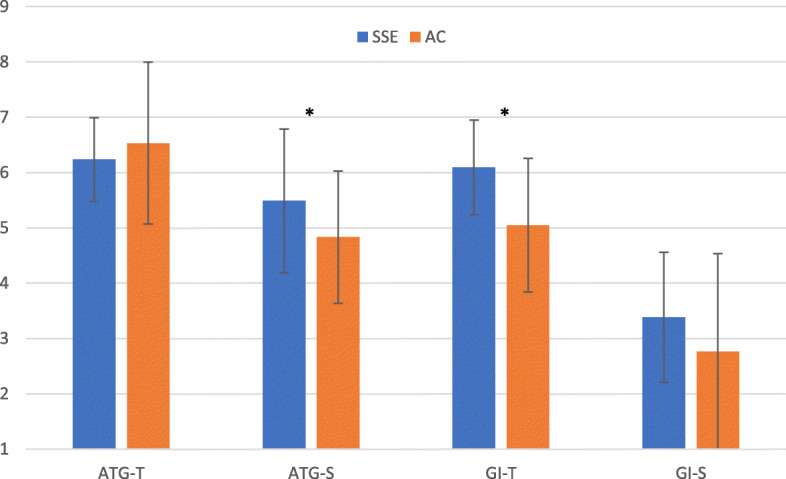
The averaged four subscale scores of the Physical Activity Group Environment Questionnaire (PAGEQ) in Square Stepping Exercise (SSE) and Active Control (AC) conditions. ATG-T: Individual Attractions to the Group—Task; ATG-S: Individuals Attractions to the Group—Social; GI-T: Group Integration—Task; GI-S: Group Integration—Social. Error bar: *SD*. *: Significant difference at *p* < .01

Figure 4 shows the averaged four PAGEQ subscale scores in the SSE and AC conditions. A one-way repeated-measures MANOVA on the PAGEQ subscale scores indicated that the main effect of conditions was significant, (*F*[1,16]= 3.05, *p* = .05, η_p_^2^ = 0.48). Separate univariate ANOVAs revealed that the scores of ATG-S (*F*[1,16] = 10.10, *p* < .01, η_p_^2^ = 0.39, *M* = 5.49, *SD* = 1.30) and GI-T (*F*[1,16] = 11.56, *p* < .01, η_p_^2^ = 0.42, *M* = 6.09, *SD* = 0.85) in the SSE condition were significantly higher, compared to the active control condition (ATG-S: *M* = 4.83, *SD* = 1.19; GI-T: *M* = 5.05, *SD* = 1.21).

## Discussion

The present study aimed to examine the effects of SSE on cognitive and social functions in sedentary young adults as there is scarce research on the SSE program with young adults. As the study was conducted amid the COVID-19 pandemic, all exercise sessions were carried out online. Therefore, the study also investigated whether group cohesion could be enhanced in the SSE condition compared to the active control condition, even though all the exercise sessions were conducted online.

Results revealed that SSE was effective to improve executive function such as abstract reasoning, mental flexibility, and problem-solving skills in sedentary young adults. Such improvement was not observed in the active control condition although the intensity and bodily movements of the two exercises were similar. The significant improvement on the MCST score observed in the present study was consistent with the finding reported by Teixeira et al. [9]. In their study, participants were 41 older adults and the 40-min SSE sessions were implemented three times per week over 16 weeks (i.e., 48 sessions in total). Given that the training period was relatively short in this study (3 sessions in a week), the significant improvement on the MCST score was somewhat surprising. However, the consistent findings of these two studies suggest that the improved executive function observed in the SSE condition might be attributed to the cognitive demands required in SSE.

Executive functions refer to a family of top-down mental processes that are required when individuals must concentrate and pay attention, when going on automatic or relying on instinct or intuition are ill-advised, insufficient, or impossible [25]. According to Diamond [25, 26], there are three core executive functions: inhibition (inhibitory control, including behavioral inhibition and interference control), working memory (holding information in mind and mentally working with that information), and cognitive flexibility (mental flexibility that enables us to flexibly adjust to changing demands or to overcome unexpected problems). In the SSE program, participants had to pay close attention to the instructor’s demonstrations to memorize stepping patterns and execute those steps subsequently. They are also required to inhibit and change their natural walking behaviors to complete the demonstrated complex steps accurately. On the other hand, participants simply followed the movements demonstrated by the instructor in the active control condition. Thus, the three core executive functions were stimulated in the SSE program, but not in the simple stepping exercise conducted in the active control condition. These unique characteristics of the SSE program are considered to contribute to the improved executive function measured by the MCST. When SSE is conducted in a group setting, it has the potential to promote social interactions [2]. In the present study, participants perceived that social interaction with their group (ATG-S) and closeness and bonding existing in their group (GI-T) were significantly higher, compared to the active control condition. This result demonstrated that social interactions and bonding were promoted more in the SSE sessions, even though participants were not physically at the same location. It was reported that social interaction among participants were enhanced in the face-to-face SSE program [10, 11]. In the online SSE sessions in the present study, interaction between participants were promoted by making a pair in every session. The partners provided encouragements through the provision of virtual hi-fives and words of encouragements whenever their partners have completed a round of the SSE exercise. These encouragements from the partners were considered to increase the participant’s perception of personal involvement with the SSE exercise and the closeness and bonding within the pair. The score of the other two PAGEQ subscales (ATG-T and GI-S) was similar between the two exercise conditions. These non-significant results make sense, given that the intensity of the exercises was similar and the duration of each session was identical. Participants had no chance to go out after the session because they joined the exercise sessions in the evening from their home under the circumstances of the COVID-19 pandemic.

In the present study, SSE was conducted online and found to be effective to enhance executive function and group cohesion in sedentary young adults. These novel approach and findings are the strengths of the present study. Despite the strengths, there are limitations to the current study. Participants were not fully randomized due to their availabilities when they were allocated to a group. Furthermore, the natural development of social interactions after the exercise sessions was inhibited under the COVID-19 pandemic circumstances.

People aged 60 years and over are more vulnerable to the coronavirus and at higher risk of developing serious illness [27]. Considering the current pandemic situation, it is worthwhile to explore the possibility of the online SSE approach to older adults and examine its long-term effects on cognitive, physical, psychological, and social functioning in both young and older adults in future research.

## Data Availability

The datasets used and/or analyzed during the current study are available from the corresponding author on reasonable request.

## References

[1] Karr JE, Areshenkoff CN, Rast P, Garcia-Barrera MA (2014). An empirical comparison of the therapeutic benefits of physical exercise and cognitive training on the executive functions of older adults: A meta-analysis of controlled trials. Neuropsychology.

[2] Shigematsu R, Okura T (2006). A novel exercise for improving lower-extremity functional fitness in the elderly. Aging Clin Exp Res.

[3] Shigematsu R, Okura T, Sakai T, Rantanen T (2008). Square-stepping exercise versus strength and balance training for fall risk factors. Aging Clin Exp Res.

[4] Shigematsu R, Okura T, Nakagaichi M, Tanaka K, Sakai T, Kitazumi S, Rantanen T (2008). Square-stepping exercise and fall risk factors in older adults: a single-blind, randomized controlled trial. J Gerontol A Biol Sci Med Sci.

[5] Ravichandran H, Janakiraman B, Yitayeh A, Sundaram S, Fisseha B (2017). Effectiveness of square stepping exercise among subjects with Parkinson’s disease: A pilot randomized controlled trial. J Geriatr Mental Health.

[6] Fisseha B, Janakiraman B, Yitayeh A, Ravichandran H (2017). Effect of square stepping exercise for older adults to prevent fall and injury related to fall: systematic review and meta-analysis of current evidences. J Exerc Rehabil.

[7] Nokham R, Kitisri C (2017). Effect of square-stepping exercise on balance in older adults: a systematic review and meta-analysis. J Phys Fit Sports Med.

[8] Pereira JR, Gobbi S, Teixeira CV, Nascimento CM, Corazza DI, Vital TM, Shigematsu R (2014). Effects of square-stepping exercise on balance and depressive symptoms in older adults. Motriz Rev Ed Fisica.

[9] Teixeira CV, Gobbi S, Pereira JR, Vital TM, Hernandéz SS, Shigematsu R, Gobbi LT (2013). Effects of square-stepping exercise on cognitive functions of older people. Psychogeriatrics.

[10] Shigematsu R, Okura T, Nakagaichi M, Nakata Y (2014). Effects of exercise program requiring attention, memory and imitation on cognitive function in elderly persons: a non-randomized pilot study. J Gerontold Geriatr Res.

[11] Noma A, Uchida R, Kurosaki T, Numao S, Nakagaichi M (2020). Effects of the Square-Stepping Exercise program on physical fitness and cognitive function in elderly. Japanese J Phys Fit Sports Med.

[12] Audiffren, M. Acute exercise and psychological functions: A cognitive-energetic approach. In: McMorris T, Tomporowski P, Audiffren M, editors, Exercise and cognitive function. Chichester, West Sussex: Wiley-Blackwell; 2009. p. 3-39.

[13] Paoletti P (2008). Crescere nell’eccellenza. [Growing in excellence].

[14] Ben-Soussan TD, Glicksohn J, Goldstein A, Berkovich-Ohana A, Donchin O (2013). Into the square and out of the box: The effects of Quadrato Motor Training on creativity and alpha coherence. Plos ONE.

[15] Ben-Soussan TD, Berkovich-Ohana A, Glicksohn J, Goldstein A (2014). A suspended act: Inceased reflectivity and gender-dependent electrophysiological change following Quadrato Motor Training. Front Psychol.

[16] Ben-Soussan TD, Avirame K, Glicksohn J, Goldstein A, Harpaz Y, Ben-Shachar M (2014). Changes in cerebellar activity and inter-hemispheric coherence accompany improved reading performance following Quadrato Motor Training. Front Syst Neurosci.

[17] Faul F, Erdfelder E, Lang AG, Buchner A (2007). G*Power 3: A flexible statistical power analysis program for the social, behavioral, and biomedical sciences. Behav Res Methods.

[18] Cohen J. Statistical power analysis for the behavioral sciences (2nd ed.). New York: Lawrence Earlbaum Associates; 1988.

[19] The Institute for Square Stepping Exercise. Samples of Square Stepping Exercise. 2020. https://square-step.org/mini_sse_patterns/. Accessed 01 May 2000.

[20] Nelson HE (1976). A modified card sorting test sensitive to frontal lobe defects. Cortex.

[21] Millisecond Software (2016). Inquisit 5 [Computer software]. Retrieved from https://www.millisecond.com.

[22] Caffarra P, Vezzadini G, Dieci F, Zonato F, Venneri A (2004). Modified Card Sorting Test: Normative data. J Clin Exp Neuropsychol.

[23] Goldberg TE, Harvey PD, Wesnes KA, Snyder PJ, Schneider LS (2015). Practice effects due to serial cognitive assessment: Implications for preclinical Alzheimer’s disease randomized controlled trials. Alzheimer’s Dementia.

[24] Estabrooks P, Carron A (2000). The physical activity group environment questionnaire: an instrument for the assessment of cohesion in exercise classes. Group Dynam Theory Res Pract.

[25] Diamond A, Ling DS. Review of the evidence on, and fundamental questions about, efforts to improve executive functions, including working memory. In: Novick J, Bunting MF, Dougherty MR, Engle RW, editors, Cognitive and working memory training: Perspectives from psychology, neuroscience, and human development. New York: Oxford University Press; 2019; p. 143-431.

[26] Diamond A (2013). Executive functions. Annu Rev Psychol.

[27] World Health Organization. (2020, October 12). Coronavirus disease (COVID-19). https://www.who.int/emergencies/diseases/novel-coronavirus-2019/question-and-answers-hub/q-a-detail/coronavirus-disease-covid-19

